# Early nursing interventions for neurogenic bowel dysfunction in acute-phase spinal cord injury patients: A non-concurrent controlled trial

**DOI:** 10.1097/MD.0000000000043912

**Published:** 2025-08-08

**Authors:** Hongyan Li, Ruiling Wang, Zhaoxuan Wang, Di Zhang

**Affiliations:** a Department of Spine Surgery, The Third Hospital of Hebei Medical University, Shijiazhuang, China.

**Keywords:** early nursing intervention, gastrointestinal management, neurogenic bowel dysfunction, quality of life, spinal cord injury

## Abstract

**Background:**

Traumatic spinal cord injury (SCI) often leads to neurogenic bowel dysfunction (NBD), causing significant gastrointestinal issues that adversely affect patients’ quality of life (QoL). The current study evaluates the effectiveness of early nursing interventions in managing NBD among SCI patients.

**Methods:**

A non-concurrent controlled trial design included 125 participants with acute-phase SCI. The control group (n = 56) received standard nursing care, while the intervention group (n = 69) underwent personalized gastrointestinal management. Data were collected via NBD scores and the Short Form-12 QoL questionnaire.

**Results:**

Participants’ average age was 41.55 ± 12.59 years, with a near-equal gender distribution. The intervention group showed significant improvement in bowel movement frequency, reduced defecation time, and decreased reliance on medication, leading to enhanced QoL compared to the control group.

**Conclusion:**

The study highlights the critical role of early nursing interventions in improving NBD outcomes for SCI patients. Future research should consider multicenter designs to enhance generalizability and focus on chronic NBD management.

## 1. Introduction

Traumatic spinal cord injury (SCI) not only results in long-term dysfunction across multiple organ systems and a decline in overall quality of life (QoL), but it also leads to significant autonomic dysfunctions, particularly impacting gastrointestinal functions.^[1,2]^ Based on pathophysiological changes the early acute phase is defined to be 2 to 48 hours after the injury, the subacute phase from 2 days to 2 weeks, and the intermediate phase from 2 weeks to 6 months.^[3]^ However, the clinically acute phase is usually defined as the first 4 to 5 weeks after the injury. Between 27% and 62% of patients with SCI report having problems with their bowel, the most frequent symptoms are obstipation, distension, and abdominal pain.^[4]^ Spinal shock leads to loss of all activities, under the level of injury, including autonomic function and reflexes. The acute phase following SCI often witnesses severe gastrointestinal dysfunction, which poses a substantial threat to the physical health of patients and adversely affects their psychological well-being and life quality.

Despite the prevalence of gastrointestinal dysfunction after SCI, there is currently no consensus on the most effective management strategies. Standard care typically involves basic gastrointestinal management practices such as dietary adjustments and scheduled defecation.^[5–7]^ In contrast, proactive care interventions may include more personalized and comprehensive strategies like transanal irrigation and abdominal massage, aimed at enhancing bowel function and reducing complications.^[8–10]^

Although numerous care interventions for neurogenic bowel dysfunction (NBD) exist, managing this condition remains challenging. Research indicates that early intervention in care significantly impacts QoL.^[11]^ However, there are few published articles on the impact of early nursing interventions on NBD following traumatic SCI. Therefore, the primary aim of this study is to explore whether early proactive nursing interventions can improve intestinal function in SCI patients and enhance their QoL.

## 2. Methods

### 2.1. Study design

This investigation employed a non-concurrent controlled trial design, where participants were allocated to control and intervention groups based on admission time periods (May–August 2023 for controls; September 2023–April 2024 for intervention group). While this design differs from randomized controlled trials, it provides a pragmatic approach for evaluating interventions in real-world clinical settings.

### 2.2. Population and data collection

This study included patients in the acute phase of SCI who were treated at the Department of Spine Surgery, The Third Hospital of Hebei Medical University, Shijiazhuang, China. Inclusion criteria included adults over the age of 18 diagnosed with acute cervical SCI, who sought medical treatment at our hospital within 2 weeks of the injury, exhibited clinical signs of intestinal dysfunction, and were fully conscious with the capability for normal communication. Their families also needed to be willing to participate in the study. Exclusion criteria were patients with non-SCI related intestinal diseases, a history of perianal diseases, severe complications during treatment requiring cessation of intestinal interventions, and those unable to cooperate with early nursing interventions.

The dataset comprised all patients aged 18 or older in the acute phase of SCI, diagnosed with NBD between May 2023 and April 2024, totaling 213 individuals. Each patient received a questionnaire, and professional nurses explained the study’s purpose; however, only 125 agreed to participate voluntarily. The study utilized a non-concurrent controlled trial design, with 56 patients admitted from May to August 2023 serving as the control group, and 69 patients admitted from September 2023 to April 2024 forming the subsequent study group. Patients were stratified based on the nursing interventions received and divided into 2 groups: (a) the control group, which received routine nursing care, and (b) the intervention group: received early active care intervention initiated shortly after admission.

In the control group, routine nursing care was administered. Upon admission, comprehensive assessments were conducted, including evaluations of spinal injury, gastrointestinal function, and medical history. Daily monitoring of bowel movements was performed, alongside dietary counseling aimed at increasing the consumption of fresh vegetables, fruits, and high-fiber foods, while reducing intake of gas-producing foods to prevent bloating. In cases of difficult defecation, prescribed oral medications or glycerin enemas were administered. Post-defecation care included maintaining cleanliness and dryness of the perianal skin.

In the intervention group, targeted interventions for gastrointestinal dysfunction were prioritized. Early nursing management emphasized dietary and lifestyle modifications following SCI, supplemented by adjunctive medications and devices to create personalized gastrointestinal management programs for each patient. Recommendations included establishing a morning bowel routine to capitalize on naturally enhanced colonic activity, initiating bowel care 20 to 45 minutes after meals to take advantage of the gastrocolic reflex, and utilizing digital anorectal stimulation along with suppositories and laxatives to facilitate the recto-colonic reflex. Effectiveness of the interventions was assessed using standardized scales, the results of which will be presented in the subsequent sections.

### 2.3. Questionnaire surveys

Questionnaire surveys were employed to gather basic demographic and functional data, including gender, age, injury level, and American Spinal Injury Association impairment scale scores. Before administering the questionnaire, 2 nurses provided participants and their family members with a brief overview of the study’s objectives to enhance compliance. Participants had the option of completing either an online or a written questionnaire, which included the NBD score and the Short Form-12 (SF-12) to collect fundamental information and assess QoL. Data collected from the questionnaires were analyzed to evaluate correlations between demographic variables, injury characteristics, and patient-reported outcomes.

NBD score is a specialized questionnaire designed for patients with SCI. It comprises 10 items addressing various issues including the frequency of bowel movements, headache, perspiration, or discomfort before or during defecation; use of tablets and drops for constipation; time spent on each defecation; frequency of digital stimulation or evacuation; fecal incontinence; medication against fecal incontinence; flatus incontinence; and perianal skin problems.^[12]^

The SF-12 is a condensed version of the original 36-item quality of life (QoL) questionnaire and is available in Chinese.^[13]^ It includes 12 items divided into a Mental Component Summary and a Physical Component Summary, with scores on each subscale ranging from 0 to 100 points. Higher scores indicate a better QoL, assessing both mental and physical health-related QoL.

Primary outcome was NBD score improvement from admission to discharge. Secondary outcomes included SF-12 QoL scores.

### 2.4. Ethical compliance

This study adhered strictly to the ethical standards outlined in the Declaration of Helsinki. Ethical approval and consent for participation were secured from the ethics committee of the Third Hospital of Hebei Medical University, approval number W2023-067-1. All participants provided written informed consent before inclusion in the study. Additionally, the study procedures were reviewed to ensure compliance with local data protection and privacy regulations.

### 2.5. Statistical analysis

Data analyses were conducted using SPSS software, version 25.0. Sample size calculation was performed using G*Power software, assuming a medium effect size (Cohen d = 0.5), two-tailed α = 0.05, and power = 80%. This calculation indicated a minimum requirement of 64 participants per group. The final sample included 125 participants (control n = 56, intervention n = 69), which exceeded the minimum requirements. The Kolmogorov–Smirnov test was applied to assess the distribution of variables. Descriptive statistics were reported as mean ± standard deviation for normally distributed data, and medians for non-normally distributed data. Categorical and nominal data were presented as frequencies and percentages. Univariable analysis was performed to evaluate the significance of associations between variables from the NBD questionnaire and the SF-12 score. Variables with significant univariable effects were further analyzed using logistic multivariate regression analysis, with odds ratios presented alongside a 95% confidence interval. Microsoft Excel was used to construct tables, with a significance level set at 0.05.

## 3. Results

### 3.1. Participant flow and safety

All 125 enrolled participants completed the study protocol. No serious adverse events related to nursing interventions were reported. The control group (n = 56) received standard care from May to August 2023, while the intervention group (n = 69) received enhanced nursing interventions from September 2023 to April 2024. The participant recruitment and allocation process are illustrated in flow diagram. Of the 213 eligible patients with acute SCI and NBD identified during the study period, 125 (58.6%) consented to participate and completed the study protocol.

### 3.2. Demographic information

The response rate for our study was 58.6% (n = 125), with participants averaging 41.55 ± 12.59 years of age. The age distribution was notably skewed towards older adults, with the largest group being those aged 50 to 59 years (32.80%), followed by 18 to 29 years (26.40%). The gender distribution was relatively balanced, with 55.20% female and 44.80% male participants. Most injuries were cervical (41.5%), followed by thoracic (29.3%).

A significant majority (89.60%) of participants were married, with the remainder (10.40%) unmarried. Over 3-quarters of the injuries (76.00%) were incomplete, with the remainder (24.00%) being complete, and most were caused by trauma (84.8%). According to the American Spinal Injury Association impairment scale, 35.20% were classified as level D (least severe), with 24.00% at level A (most severe), 20.80% at level B, and 20.00% at level C. Educational attainment varied, with 44.80% having completed high school, 17.60% primary school, 16.00% junior middle school, 6.40% holding a Bachelor’s degree, and 15.20% attaining higher educational levels.

The average hospital stay was 20.07 ± 3.47 days, with the control group averaging 20.11 ± 3.44 days and the intervention group slightly lower at 20.04 ± 3.52 days; however, this difference was not statistically significant. Body mass index analysis revealed that 42.40% of participants were obese, 31.20% overweight, 24.00% normal weight, and 2.40% underweight, as detailed in Table 1.

**Table 1:** Demographic and clinical characteristics of the study participants.

Category	N	%
Age (yr)		
18–29 yr	33	26.40%
30–39 yr	24	19.20%
40–49 yr	27	21.60%
50–59 yr	41	32.80%
Gender		
Male	56	44.80%
Female	69	55.20%
Marriage		
Unmarried	13	10.40%
Married	112	89.60%
ASIA		
A	30	24.00%
B	26	20.80%
C	25	20.00%
D	44	35.20%
Education status		
Primary school	22	17.60%
Junior middle school	20	16.00%
High school	56	44.80%
Bachelor	8	6.40%
Higher	19	15.20%
Severity of injury		
Complete	30	24.00%
Incomplete	95	76.00%
Causes of injury		
Car	69	55.2%
Fall	37	29.6%
Other	19	15.2%
Hospital stay (day)		
	20.07 ± 3.47	
BMI		
Underweight	3	2.4%
Normal	30	24.0%
Overweight	39	31.2%
Obesity	53	42.4%
QOL		
NBD on admission	48.36 ± 30.02	
NBD on discharge	49.34 ± 29.92	
SF-12 on admission	49.33 ± 29.88	
SF-12 on discharge	50.62 ± 30.70	

ASIA = American Spinal Injury Association score, BMI = body mass index, NBD = neurogenic bowel dysfunction (NBD) score, QOL = quality of life, SF-12 = Short Form-12.

### 3.3. Outcomes of bowel management

As shown in Table 2, both control and intervention groups displayed minimal differences across several NBD indicators at admission. For instance, the frequency of bowel movements varied slightly between daily, 2 to 6 times per week, and less than once per week across both groups with no statistically significant differences observed. Similarly, the time spent per defecation and symptoms related to defecation such as headache, perspiration, or discomfort showed no significant disparities. The intervention group showed a notable distinction only in the reduced frequency of medication used for treating fecal incontinence.

**Table 2:** Comparison of neurogenic bowel dysfunction (NBD) scores between control and intervention groups upon admission.

Items of the NBD questionnaire	Control group	Intervention group	χ²	*P* value
Frequency of bowel movements				
Daily	17	23	0.25	.88
2–6 times per week	18	23		
Less than once per week	21	23		
Time used for each defecation				
<30 min	19	23	0.45	.80
31–60 min	15	22		
More than an hour	22	24		
Symptoms of headache, perspiration, or discomfort before or during defecation				
No	29	29	0.26	.61
Yes	27	40		
Taking medication (tablets) to treat constipation				
No	28	30	0.30	.58
Yes	28	39		
Taking medication (drops or liquid) to treat constipation				
No	22	30	0.70	.40
Yes	34	34		
Frequency of digital stimulation				
Less than once per week	32	41	0.01	.94
Once or more per week	24	28		
Frequency of involuntary defecation				
Daily	9	14	0.67	.88
1–6 times a week	14	17		
3–4 times a month	16	16		
A few times a year or less	17	22		
Taking medication to treat fecal incontinence				
No	21	45	8.45	.00
Yes	35	24		
Having experienced uncontrollable flatus				
No	28	37	0.05	.82
Yes	28	32		
Having perianal skin problems				
No	30	35	0.02	.89
Yes	26	34		

At discharge, the results illustrated a marked improvement in the intervention group across most evaluated parameters, suggesting that the interventions were effective. Notably, the daily frequency of bowel movements in the intervention group increased significantly, with a higher percentage of patients managing bowel movements in <30 minutes: an improvement underscored by a significant *P* value (*P* < .01). Additionally, the intervention group reported fewer symptoms of discomfort associated with defecation, indicating enhanced comfort and reduced autonomic dysreflexia. This was complemented by a substantial reduction in the usage of both tablets and liquid medication for constipation, pointing towards a decreased dependency on pharmacological aids post-intervention.

Further, the frequency of digital stimulation needed less than once per week showed significant improvement, which correlates with fewer instances of involuntary defecation: a crucial factor for patient dignity and QoL. This reduction in involuntary defecation and the significant decrease in patients experiencing uncontrollable flatus or perianal skin problems post-intervention reveal the profound positive effects of the targeted clinical measures.

In synthesizing these findings, it becomes evident that the QoL for patients with NBD can be considerably enhanced through specific clinical interventions. The data indicates that less time consumed during defecation and reduced symptoms of autonomic dysreflexia are key factors that contribute to an improved QoL. Moreover, the decrease in medication use, both in terms of frequency and type (drops/liquid vs tablets), further suggests that more natural defecation processes could be reestablished through the interventions.

## 4. Discussion

NBD is increasingly recognized as a critical factor affecting the QoL and reintegration into the community for individuals with SCI.^[14–17]^ Recent studies highlight that NBD often has a more significant impact on QoL than bladder function, wheelchair use, or pain. However, research on NBD among Chinese individuals with SCI remains limited. So, this study aimed to highlight the management of NBD and its impacts on people with SCI who received treatment at The Third Hospital of Hebei Medical University from June 2023 to January 2024.

**Figure 1.**
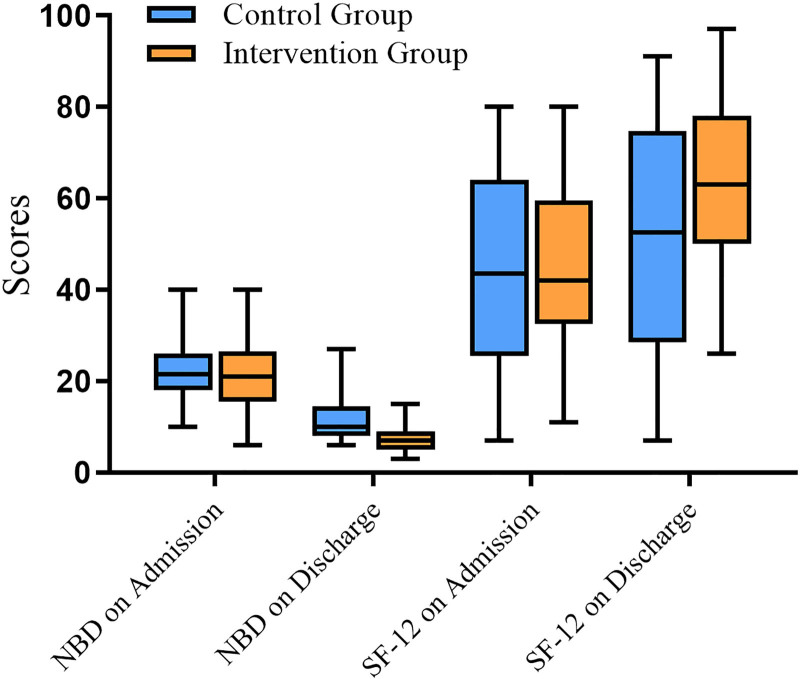
The comparison of NBD and SF-12 scores at admission and discharge between control and intervention groups. NBD = Neurogenic bowel dysfunction, SF-12 = Short Form-12.

Active nursing interventions significantly improved scores on the NBD and SF-12, as depicted in Figure 1. Constipation is often linked to a lack of routine and infrequent management practices.^[18]^ In this study, the frequency of bowel movements and the duration of each defecation were closely associated with health-related scores. Previous research has indicated that longer defecation times correlate with poorer QoL.^[19,20]^ Initially, there was no significant difference in the time used for each defecation among participants at admission; however, following proactive nursing interventions, which included early nursing management focusing on dietary and lifestyle modifications post-SCI, supplemented by adjunct medications and devices, personalized gastrointestinal management programs were developed for each patient. Recommendations included establishing a morning bowel routine to leverage naturally enhanced colonic activity, initiating bowel care 20 to 45 minutes after meals to utilize the gastrocolic reflex, and employing digital anorectal stimulation alongside suppositories and laxatives to facilitate the recto-colonic reflex. As a result, the intervention group showed significant improvements in the duration of each defecation and frequency of bowel movements compared to the control group. Even the standard care group showed significant improvements compared to their baseline at admission.

For post-SCI intestinal dysfunctions and related issues, home care interventions are also crucial for enhancing QoL of patients.^[21–23]^ Our nursing intervention strategies focused on active family involvement. This approach influenced the management of intestinal dysfunctions, such as impacting the use of medications (no matter tablets or liquids). In the intervention group, there was a noticeable reduction in medication use, as shown in Table 3, aligning with previous research findings.^[24]^ The frequency of digital stimulation in the intervention group was at least once per week, significantly better than that in the control group.

**Table 3:** Comparison of neurogenic bowel dysfunction (NBD) scores between control and intervention groups at discharge.

Items of the NBD questionnaire	Control group	Intervention group	χ²	*P* value
Frequency of bowel movements				
Daily	35	46	25.38	*P* < .01
2–6 times per week	5	23		
Less than once per week	15	0		
Time used for each defecation				
<30 min	32	45	10.56	.01
31–60 min	16	24		
More than an hour	8	0		
Symptoms of headache, perspiration, or discomfort before or during defecation				
No	49	9	65.94	*P* < .01
Yes	7	60		
Taking medication (tablets) to treat constipation				
No	13	56	39.66	*P* < .01
Yes	43	13		
Taking medication (drops or liquid) to treat constipation				
No	11	55	42.38	*P* < .01
Yes	45	14		
Frequency of digital stimulation				
Less than once per week	39	69	21.73	.00
Once or more per week	17	0		
Frequency of involuntary defecation				
Daily	9	0	35.94	.00
1–6 times a week	0	14		
3–4 times a month	20	6		
A few times a year or less	27	49		
Taking medication to treat fecal incontinence				
No	44	69	13.98	.00
Yes	12	0		
Having experienced uncontrollable flatus				
No	13	58	44.19	.00
Yes	43	11		
Having perianal skin problems				
No	49	69	6.93	.01
Yes	7	0		

We observed that improvements in the frequency of involuntary defecation were associated with enhancements in patient QoL. This correlation may be linked to psychological factors, notably affecting self-esteem, aligning with findings from previous studies.^[25]^

Significant amelioration of perianal skin issues was evident in both groups, with the intervention group showing more pronounced resolution. We attribute this primarily to the direct impact of proactive nursing interventions on the condition of the perianal skin, and secondarily to the mitigation of the detrimental effects of intestinal dysfunctions on perianal skin integrity. Overall, these improvements played a significant role in enhancing patient QoL.

### 4.1. Limitations

This study focused solely on patients in the acute phase of SCI, yet many individuals experiencing chronic intestinal dysfunctions present more severe complications and warrant attention in nursing practices. Future research aims to include patients with chronic intestinal dysfunctions due to SCI. Although this study was conducted at a spinal surgery center, all participants were recruited from a single center, thus the sample may not represent the broader population but only reflects Han Chinese patients with NBD following SCI in North China. Some participants were unable to complete the assessments independently and required family assistance, inevitably introducing some bias. Future studies should address this issue, potentially by collaborating with other trauma emergency centers or rehabilitation departments to conduct large-scale, multicenter research, thereby providing more comprehensive insights into the care of neurogenic intestinal tracts. The non-randomized design represents a key limitation, as temporal allocation may introduce confounding factors and limits causal inference compared to randomized controlled trials. This study was not prospectively registered in a clinical trial registry, which represents a limitation of the current work.

## 5. Conclusion

Active nursing interventions significantly enhance the management of NBD in individuals with SCI, thereby improving their QoL. The study demonstrated that proactive and personalized gastrointestinal management, including dietary and lifestyle modifications, use of adjunct medications and devices, and structured bowel routines, led to notable improvements in defecation frequency, duration, and QoL measures. Furthermore, involving family members in the management process contributed to reduced medication use and better outcomes. The findings highlight the importance of early and individualized interventions for improving patient well-being, aligning with prior research on the impact of bowel management on QoL for SCI patients.

## Acknowledgments

The authors express their sincere gratitude to all patients and their families who participated in this study. We acknowledge the nursing staff at the Department of Spine Surgery, The Third Hospital of Hebei Medical University, for their dedicated assistance in patient care and data collection. Special thanks to the research coordinators who facilitated the study implementation and the biostatisticians who provided guidance on data analysis. We also thank the Ethics Committee of The Third Hospital of Hebei Medical University for their review and approval of this study protocol.

## Author contributions

**Conceptualization:** Hongyan Li, Di Zhang.

**Data curation:** Hongyan Li, Ruiling Wang.

**Formal analysis:** Hongyan Li, Zhaoxuan Wang.

**Funding acquisition:** Hongyan Li.

**Investigation:** Hongyan Li, Ruiling Wang, Zhaoxuan Wang.

**Methodology:** Hongyan Li.

**Project administration:** Hongyan Li.

**Resources:** Ruiling Wang.

**Supervision:** Di Zhang.

**Visualization:** Di Zhang.
